# False Positives in Artificial Intelligence Prioritization Software for Intracranial Hemorrhage Identification in the Postoperative Period: A Report of Two Cases

**DOI:** 10.7759/cureus.44215

**Published:** 2023-08-27

**Authors:** Osmay Cardoso, Marco Adly, Mohamad Hamade, Khushi Saigal, Gaurav Saigal

**Affiliations:** 1 Radiology, University of Miami Miller School of Medicine, Jackson Memorial Hospital, Miami, USA; 2 Radiology, University of Florida College of Medicine, Gainesville, USA; 3 Radiology, University of Miami, Miami, USA

**Keywords:** acute hemorrhagic stroke, grade iv glioblastoma, giant pituitary macroadenoma, acute care surgery and trauma, neuro-surgery, artificial intelligence in healthcare, ai in stroke, intracranial hemorrhage (ich), ai and robotics in healthcare, artificial intelligence in radiology

## Abstract

The implementation of artificial intelligence (AI) in radiology has shown significant promise in the identification of acute intracranial hemorrhages (ICHs). However, it is crucial to recognize that AI systems may produce false-positive results, especially in the postoperative period. Here, we present two cases where AI prioritization software erroneously identified an acute ICH on a postoperative non-contrast CT. These cases highlight the need for a more careful radiology review of AI-flagged images in postoperative patients to avoid further unnecessary imaging and unwarranted concerns from radiologists, clinicians, and patients.

## Introduction

Artificial intelligence (AI)-powered tools have emerged as valuable aids in radiology, enhancing diagnostic accuracy and expediting patient care [1,2]. In particular, AI algorithms designed for intracranial hemorrhage (ICH) identification have garnered considerable attention due to their potential to accelerate diagnosis and improve patient outcomes [2,3]. However, it is essential to remain vigilant regarding the limitations of these AI tools, especially in the inpatient postoperative setting, where accuracy is lower and where they produce the highest false-positive results [4-6]. Here, we present two cases in our institution in which the AI prioritization tool falsely reported an acute ICH on post-surgical non-contrast CTs.

## Case presentation

First case

**Figure 1:**
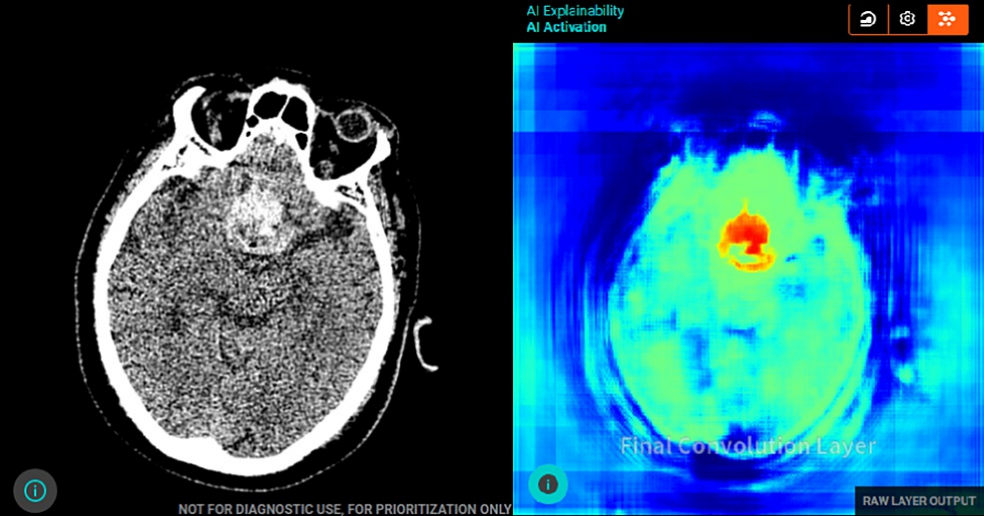
Axial non-contrast CT of the brain with AI tool analysis. (a) Shows a hyper-dense area in the frontal pituitary region that is concerning for an acute intra-cerebral hemorrhage as per AI analysis. (b) Shows the final convolution layer and feature map that the AI tool uses to identify the ICH. AI, artificial intelligence; ICH, intracranial hemorrhage.

The first case is a 69-year-old male with a history of colon cancer status post-treatment, hypertension, and bitemporal hemianopsia due to a worsening pituitary macroadenoma. The patient underwent a planned endoscopic endonasal trans-sphenoidal resection of the pituitary tumor and was admitted as an inpatient. Postoperatively, the patient had a non-contrast CT to evaluate the surgical bed. The AI prioritization software flagged the CT images as having an acute ICH as seen in Figure 1.

**Figure 2:**
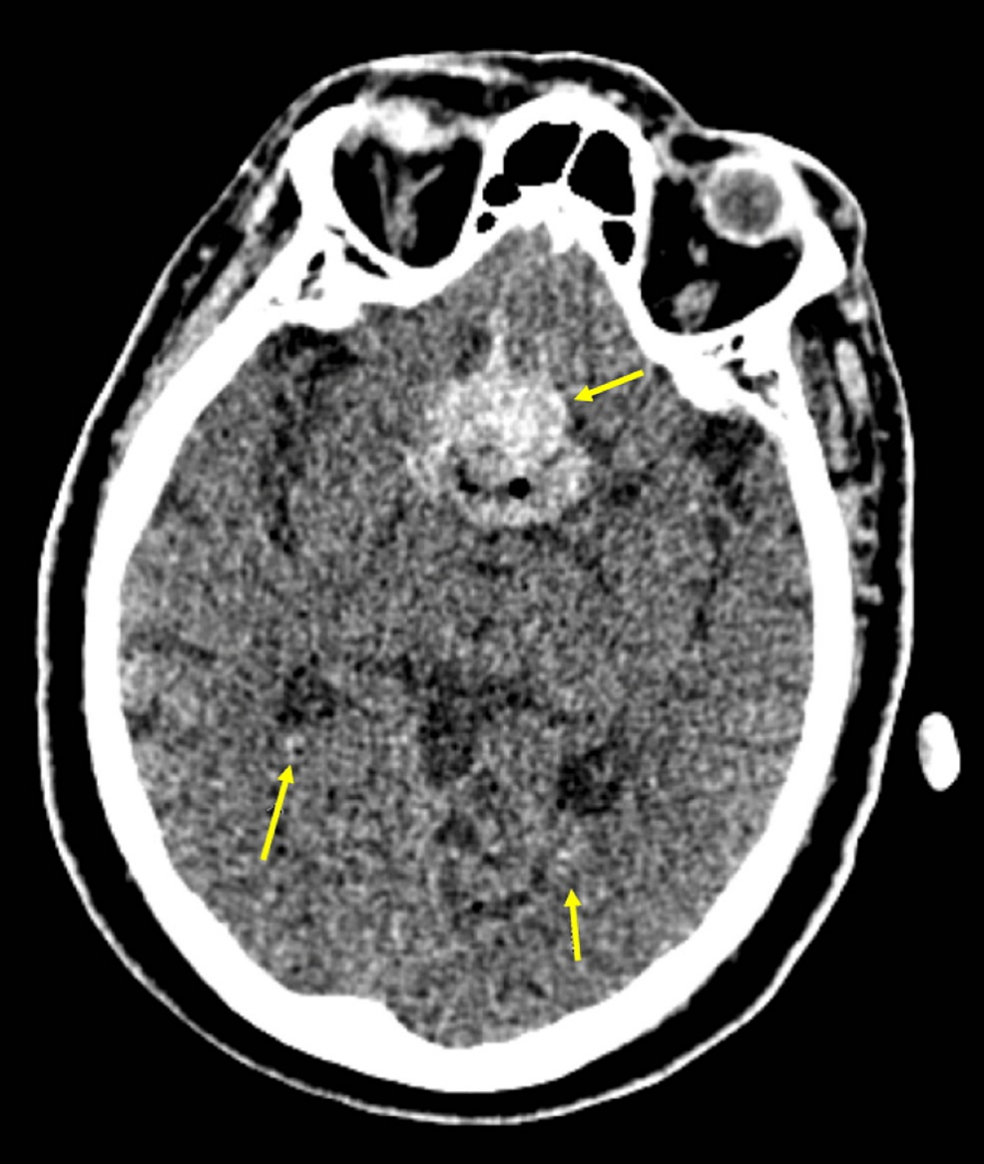
Axial non-contrast CT of the brain analyzed by the attending radiologist. The image shows postoperative changes related to a recent trans-sphenoidal pituitary macroadenoma resection (seen by the arrows). There is packing material and blood in the surgical bed. No active extravasation of blood in the surgical bed. There are small intraventricular hemorrhages versus evolving blood products (HU of 43) in the bilateral posterior horns of the lateral ventricles (seen by the arrows), and these were seen on multiple slices of the CT.

Upon radiology review, the findings were correctly changed and identified as postoperative changes, showing packing material in the surgical bed without any active extravasation of blood. Additionally, there were mild intraventricular hemorrhages or evolving blood products in the bilateral posterior horns of the lateral ventricles, which the AI software failed to detect, as seen in Figure 2. This led to a false-negative AI result being correctly picked up by a skilled radiology review.

Second case

**Figure 3:**
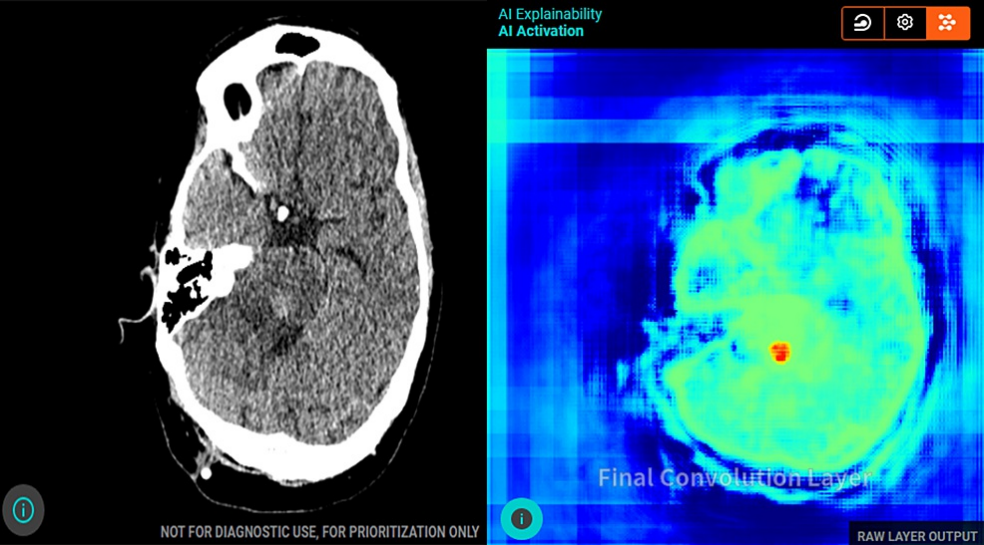
Axial non-contrast CT of the brain with AI tool analysis. (a) Shows a hyperdense area in the posterior pons that is concerning for an acute intracerebral hemorrhage as per AI analysis. (b) Shows the final convolution layer and feature map that the AI tool uses to identify the ICH. AI, artificial intelligence; ICH, intracranial hemorrhage.

The second case is an 18-year-old male with a past medical history of stereotactic needle biopsy proven grade 4 midline pontine glioma status post chemotherapy, presenting with concerns for disease progression, as well as a worsening communicating hydrocephalus that needed prompt intervention. The patient underwent surgery with the placement of a frontal Ommaya and a right parietal ventriculoperitoneal shunt. The patient had a non-contrast CT for post-surgical evaluation in which the AI software, again, incorrectly labeled the images as depicting an acute ICH as seen in Figure 3.

**Figure 4:**
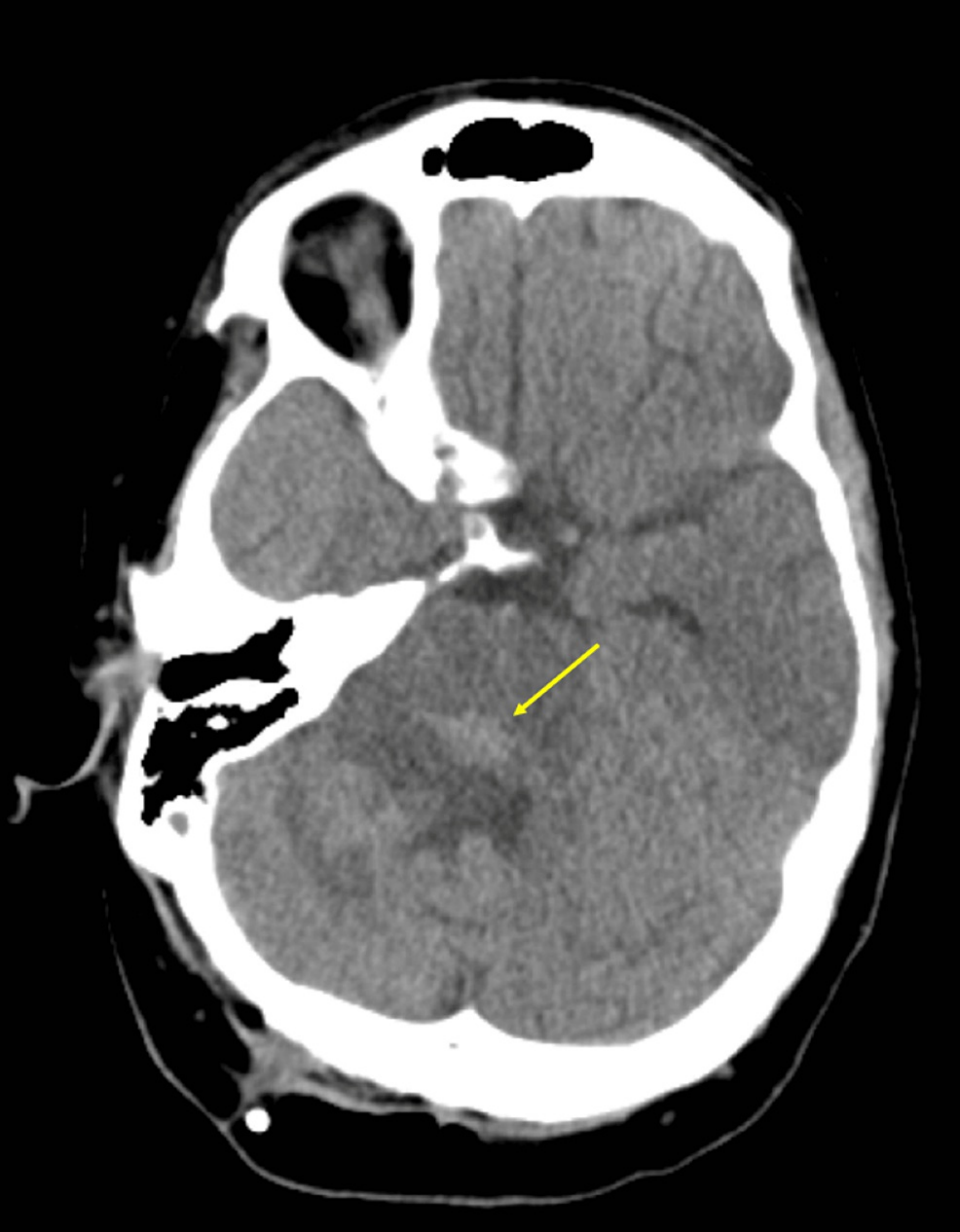
Axial non-contrast CT of the brain analyzed by the attending radiologist. The image shows post-surgical changes after a right parietal ventriculostomy catheter placement with mildly dilated ventricles. There is a focus on ill-defined hyperattenuation noted in the posterior pons (seen by the arrow), which corresponds to an area of hypercellularity seen on the follow-up MRI (area of diffusion restriction).

However, after a radiology review, the suspected ICH was deemed to be post-surgical changes with a focus on ill-defined hyperattenuation, which likely represented an area of hypercellularity secondary to the known malignancy as seen in Figure 4.

**Figure 5:**
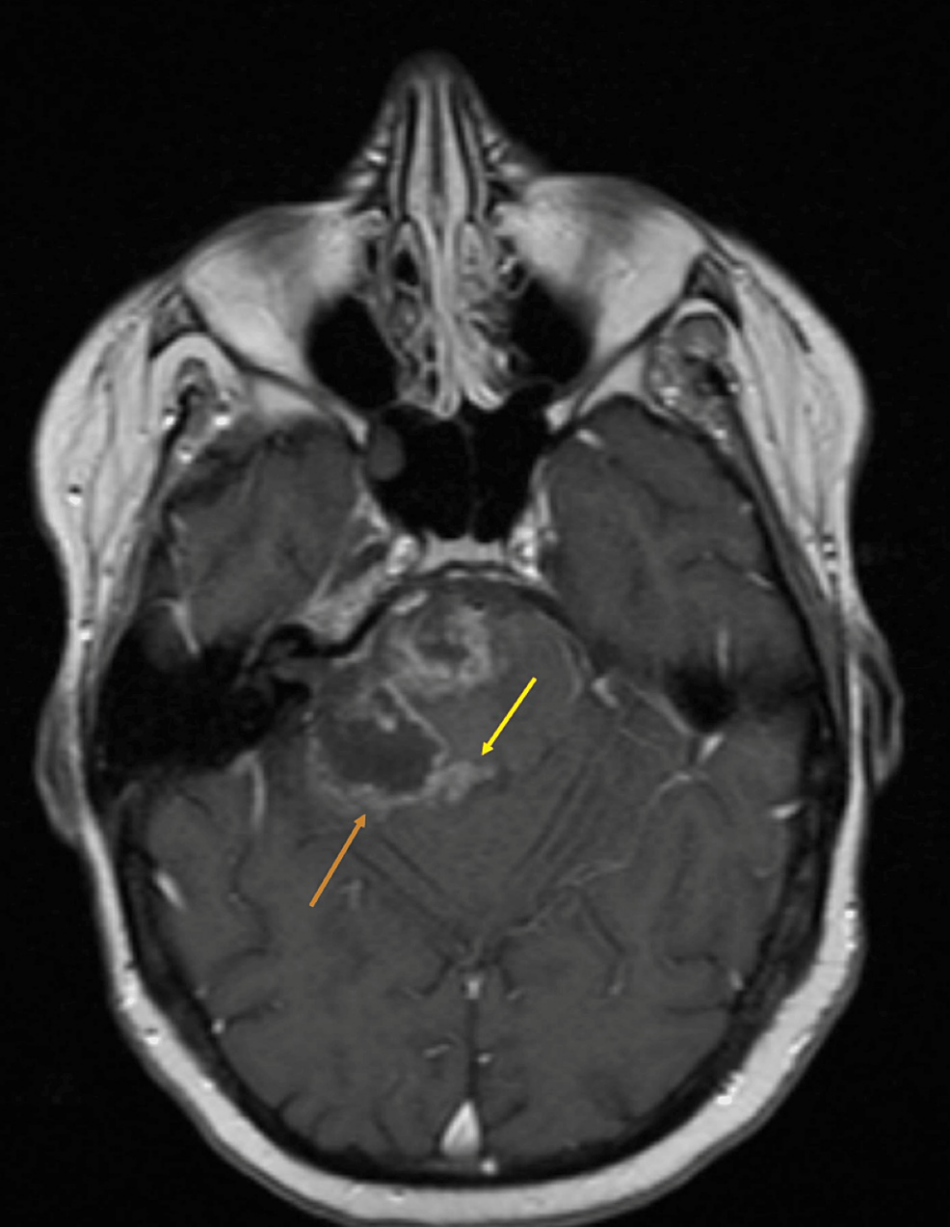
Axial T1 post-contrast MRI of the brain done to follow up previous non-contrast CT. The image shows an irregular area of rim enhancement with central low signal intensity secondary to high-grade tumor necrosis and known post-surgical changes (seen by the orange arrow). Additionally, there is also a midline region of nodular enhancement which may suggest tumor remnant/recurrence; this finding corresponds to the hyperdensity previously noted on CT (seen by the yellow arrow).

These findings were confirmed on a follow-up contrast MRI as seen in Figure 5.

## Discussion

The cases presented here illustrate the challenges of using AI prioritization software for ICH identification in patients in the postoperative period. While AI tools offer valuable support to clinicians and radiologists, they can produce false positives due to their inherent limitations in interpreting complex post-surgical changes without proper background command and patient data [1]. Although both these cases are from a single institution using a recent widespread implementation of Aidoc, similar cases have been reported in the literature, with post-surgical cases accounting for up to 24% of all AI false positives [4]. 

Clinicians must remain cautious and understand that AI interpretations should be complemented by a skilled radiology review to prevent unnecessary imaging, additional testing, and unwarranted concerns. However, even after a thorough radiology review, additional testing may still be ordered to ensure that the AI read was indeed a false positive - as was the circumstance in Case 2. These confirmatory tests add unnecessary costs and diagnostic time, especially in an otherwise simple postoperative evaluation. A recent study by Bernsteinet al. showed that false-positive rates among radiologists increased after incorrect results or suggestions by AI [7]. These radiologists also demonstrated incorrect follow-up decisions such as additional unnecessary imaging [7]. 

It is important to note that the integration of AI algorithms for ICH detection, especially in the ED, brings numerous benefits to patient care, such as rapid triage, improved efficiency, enhanced resource management, and improved outcomes [1,3,4]. Nevertheless, a more careful review and further research need to be conducted in the implementation and widespread use of these AI algorithms in inpatient postoperative patients. 

## Conclusions

The increasing use of AI in radiology, particularly in ICH identification, has demonstrated a huge potential to enhance patient care. However, it is crucial to recognize the limitations of AI algorithms, especially in the inpatient postoperative period, where false-positive results may occur more frequently. Radiology review remains essential to validate AI findings and to ensure accurate diagnoses, thus preventing unnecessary but avoidable investigations and distress. Continued collaborative efforts between AI and radiologists will optimize and ultimately minimize these false positives and enhance AI over time.
